# The external metastasis of the central nerve system germ cell tumors: case report and review of the literature

**DOI:** 10.1186/s41016-021-00246-0

**Published:** 2021-06-02

**Authors:** Peng Wang, Yanong Li, Xiaoguang Qiu

**Affiliations:** Department of Radiation Oncology, Beijing Tiantan Hospital, Capital Medical University, 119, South 4th Ring West Road, Fengtai District, Beijing, China

**Keywords:** Chemotherapy, External metastasis, Germ cell tumor, Radiotherapy

## Abstract

**Background:**

Central nervous system germ cell tumors (CNS GCTs) represent a class of rare tumors that exhibit region-specific prevalence in some Asian areas (15.3%), higher than that in North America (3.6%), and age-specific prevalence in children and adolescents. According to the 2016 World Health Organization (WHO) classification, CNS GCTs can be categorized into germinomas and non-germinomatous GCTs (NGGCTs). Owing to the compression of the interventricular foramen by enlarged GCTs in the pineal gland, the resultant obstructive hydrocephalus may result in high intracranial pressure (HIP) at an alarming pace, which urgently requires a ventriculoperitoneal shunt for the relief of severe HIP. Although CNS GCT cells tend to migrate through the cerebrospinal fluid (CSF) starting from the subependymal lining, metastasis along the ventriculoperitoneal shunt tube is extremely rare.

**Case presentation:**

In this study, we reported two cases of iGCTs with intraperitoneal metastasis. Both patients underwent ventriculoperitoneal shunt placement to alleviate HIP, and both received standard radiotherapy and chemotherapy, but they still developed abdominal metastasis, and all the abdominal masses were pathologically confirmed to be iGCTs.

**Conclusions:**

We performed a literature study and found that from 1979 to 2020, a total of 18 cases of iGCTs were metastasized outside the nervous system. We also found a shift of the median of 13.5 months and that the most common primary site was the pineal region (83.3%); moreover, nearly half of the patients (44%) died within 1 year of metastasis, indicating a poor prognosis after celiac metastasis.

**Supplementary Information:**

The online version contains supplementary material available at 10.1186/s41016-021-00246-0.

## Background

Central nervous system germ cell tumors (CNS GCTs) represent a class of rare tumors that exhibit region-specific prevalence in some Asian areas (15.3%), higher than that in North America (3.6%), and age-specific prevalence in children and adolescents [1]. According to the 2016 World Health Organization (WHO) classification, CNS GCTs can be categorized into germinomas and non-germinomatous GCTs (NGGCTs) [2]. Owing to the compression of the interventricular foramen by enlarged GCTs in the pineal gland, the resultant obstructive hydrocephalus may result in high intracranial pressure (HIP) at an alarming pace, which urgently requires a ventriculoperitoneal shunt for the relief of severe HIP. Although CNS GCT cells tend to migrate through the cerebrospinal fluid (CSF) starting from the subependymal lining, metastasis along the ventriculoperitoneal shunt tube is extremely rare [3].

## Case presentation

### Case 1

**Fig. 1:**
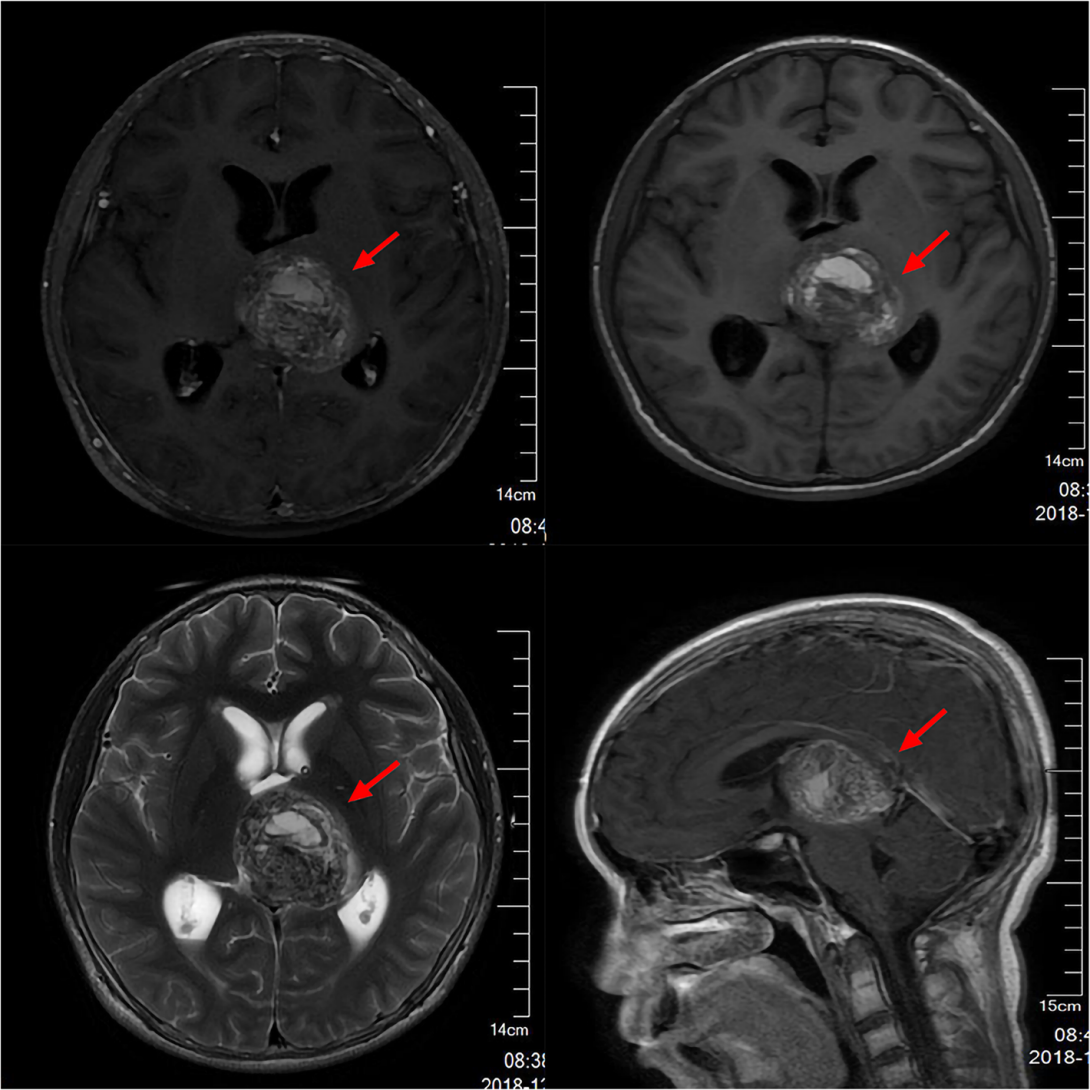
An MRI scan revealed a tumor arising from the pineal region involving the left thalamus with hydrocephalus; the tumor is marked with a red arrow

A 13-year-old Asian male presented with unsteady gait for 4 months and complained of headache, nausea, a drooping left eyelid, blurred vision for 11 days, and convulsions for 3 days. The magnetic resonance imaging (MRI) scan (Fig. 1) revealed a mass occupying the pineal gland and HIP. Alpha-fetoprotein (AFP) and chorionic gonadotropin β (β-HCG) levels in the serum were 188.2 ng/ml (0–7 ng/ml) and 716 mIU/ml (0–2.6 IU/ml), respectively, while AFP and β-HCG levels in the CSF were 5.9 ng/ml (0–7 ng/ml) and 542.9 mIU/ml (0–2.6 IU/ml). The child received a ventriculoperitoneal (VP) shunt for the relief of HIP. In accordance with the SIOP CNS GCT 96, all patients with α-AFP > 25 ng/ml or β-hCG > 50 mIU/ml levels in the serum or CSF were diagnosed with non-germinomatous germ cell tumors (NGGCTs) [3, 4]. After establishing the diagnosis, the patient initially received two cycles of platinum-based chemotherapy followed by tumor resection and completed another two courses of chemotherapy using the same regimen. Although the postoperative MRI examination showed that the tumor was completely resected and there was no metastasis, 3 months later, the patient developed spinal cord dissemination and metastasis. Craniospinal irradiation (CSI) was given after chemotherapy at a dose of 30.6 Gy/17 f intensity-modulated radiotherapy (IMRT) and a boost of 23.4 Gy/13 f IMRT. Unexpectedly, the boy reported cough alongside pain in the right costal margin and the right clavicle 8 months after placement of the VP shunt, accompanied by elevated serum AFP and β-HCG levels of 873.3 ng/ml and 742.1 mIU/ml, respectively. Whole-body 18-fluorodeoxyglucose positron emission tomography/computed tomography (18F-FDG PET/CT) showed multiple intra-abdominal foci of increased metabolic activity measured by a maximum standardized uptake value (SUVmax) of 9.8–23.1 (Fig. 4a). The patient subsequently underwent abdominal mass resection, and rapid freezing pathology during the operation proved the following immunohistochemical results: CK (+), AFP (+), HCG-β (+), Ki-67 (+), Syn (+), SMA (+), and CD30 (+). Combining the immunohistochemical results with the judgment under light microscopy, the pathologist determined that the abdominal mass in the patient was a metastasis of iGCTs (Fig. S2). Based on the combined imaging findings and laboratory results, as well as the patient having no other history except for a brain tumor, extensive intraperitoneal metastasis (EIM) of CNS GCTs was considered.

### Case 2

**Fig. 2:**
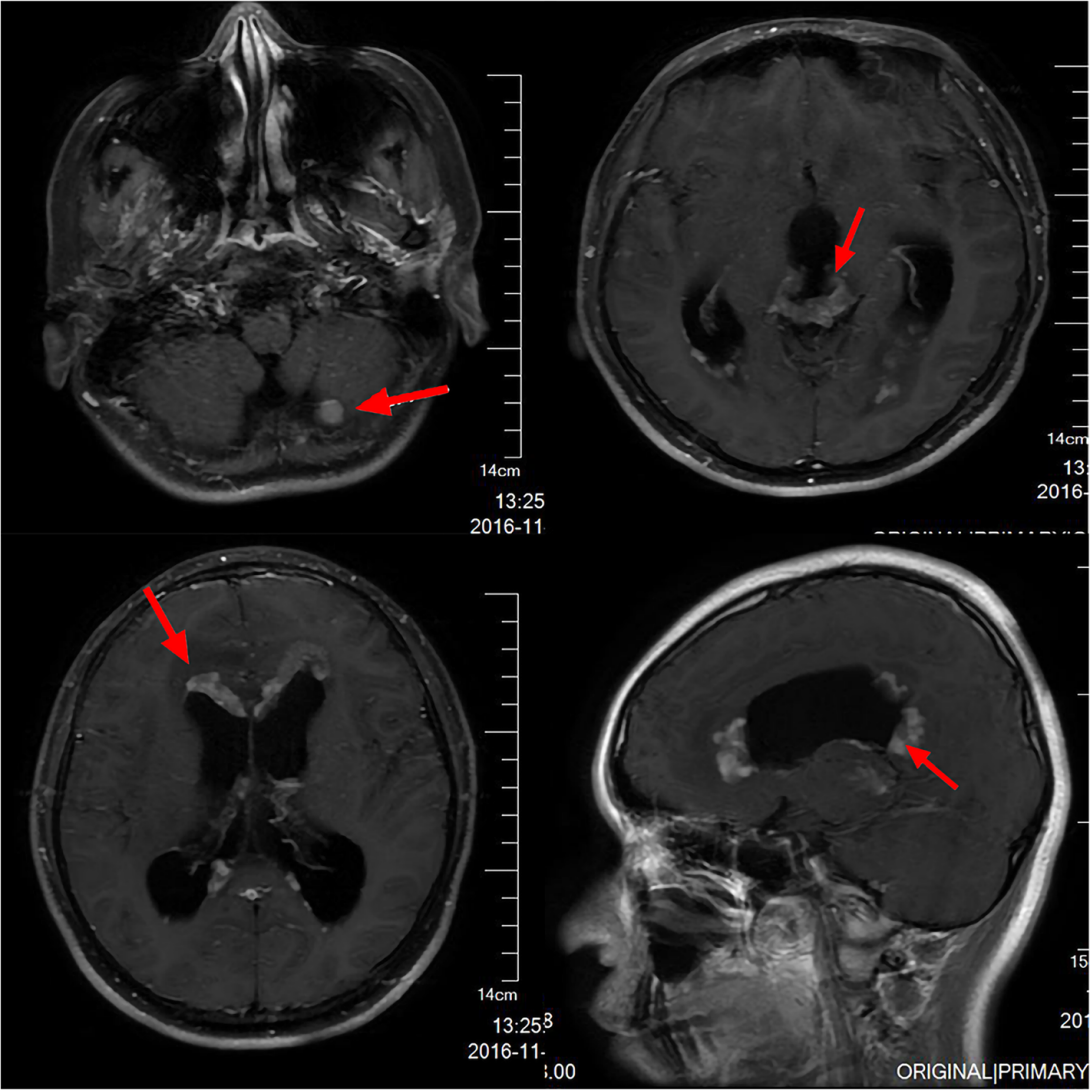
An MRI examination revealed hydrocephalus due to multiple relative space-occupying lesions in the pineal region, suprasellar region, lateral ventricles, and cerebellum (red arrow marks the lesions)

**Fig. 3:**
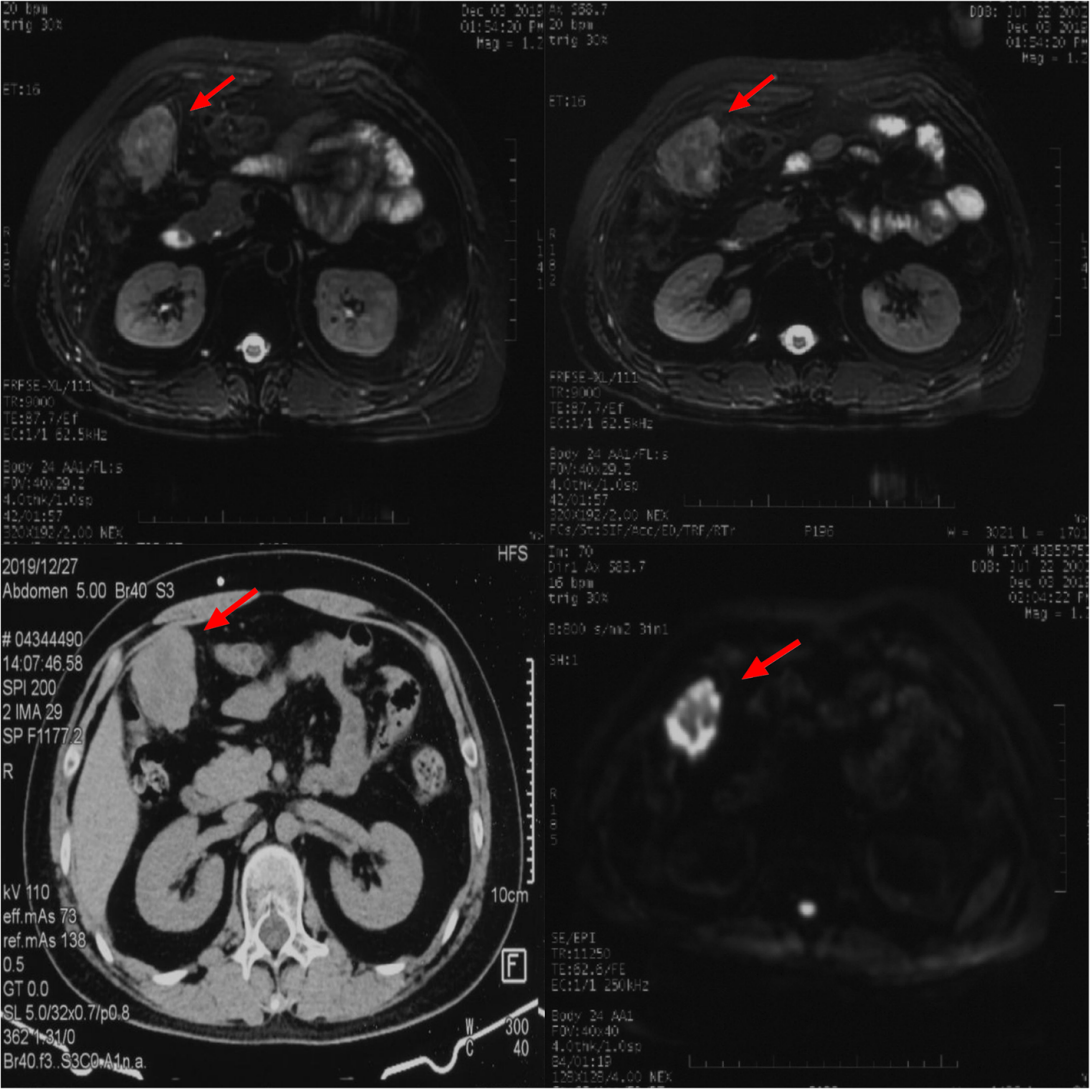
Contrast-enhanced MRI revealed rough-edged masses in the right upper abdominal mesentery with hyperecho and obvious heterogeneous enhancement. The red arrow shows the abnormal enhancement in this patient’s abdominal MRI

**Fig. 4:**
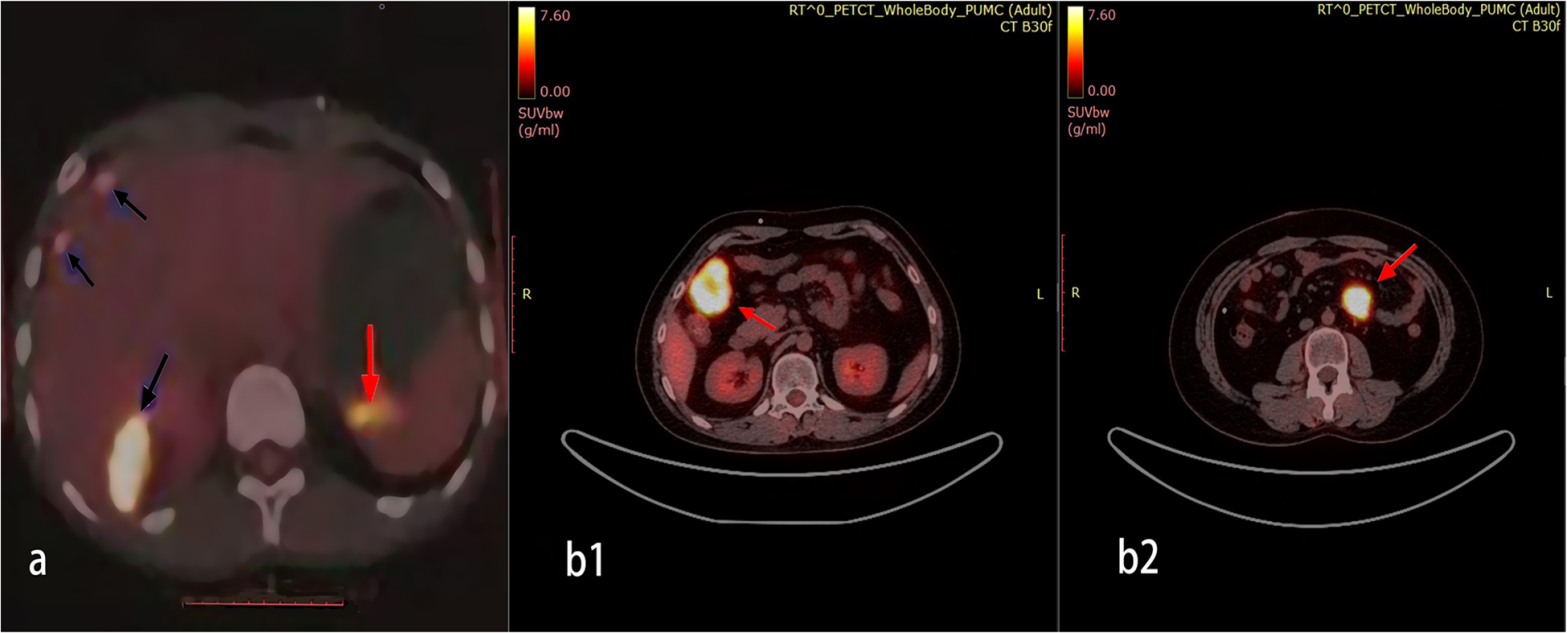
There were abnormal metabolic nodules near the inferior vena cava and the diaphragmatic apex, small nodules with slightly increased metabolism at the anterior right costal diaphragmatic angle, and extensive nodules with increased metabolism in the peritoneum (**a**) (marked in red and black arrows). Multiple-site SUV elevation in the abdominal cavity. The two main metabolic enhancement foci are concentrated in the right upper quadrant (**b1**, **b2**)

**Fig. 5:**
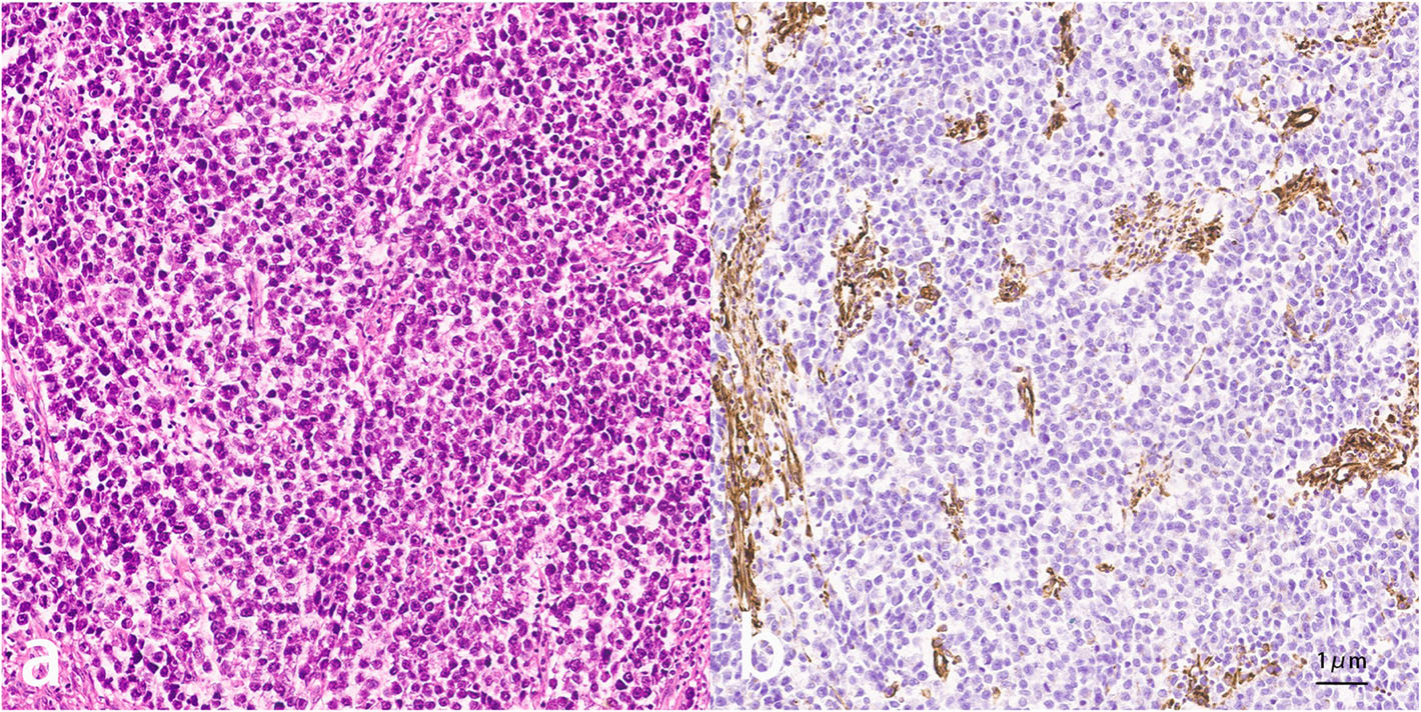
Pathological findings (**a**) and IHC analysis (**b**) of the abdominal lesion in the second patient (40x)

A 17-year-old Asian male presented with a long-standing history of generalized headache, diabetes insipidus, and intermittent fever without obvious inducement. An MRI examination (Fig. 2) revealed hydrocephalus due to space-occupying lesions, so the patient received a VP shunt to reduce HIP. Moreover, serum AFP tests were negative, but the β-HCG level in the cerebrospinal fluid reached 175.4 mIU/ml. Based on the imaging features and his symptoms, we speculated that this young man had iGCTs with intraventricular dissemination. Therefore, he received two cycles of a platinum-based regimen. Tests of the two blood tumor markers were negative, and MRI revealed 95% disappearance of the tumor after chemotherapy. Then, the patient completed irradiation of the whole central nervous system at a dose of 40 Gy/25 f. After 32 months of chemoradiation therapy, he reported intermittent abdominal pain without obvious inducement. Therefore, the abdomen of the young man was examined, and a mass in the right upper abdomen was discovered. Tests of the blood tumor markers were still negative, and MRI of the central nervous system showed no evidence of tumor recurrence (Fig. S1). However, rough-edged masses adjacent to the liver area were detected on abdominal MRI (Fig. 3). The patient then underwent a PET-CT examination, which showed multiple-site SUV elevation (12.1 to 13.6) in the abdominal cavity, so abdominal metastasis of GCTs was considered (Fig. 4 b1, b2). Therefore, urgent tumor resection was performed, and the returned pathological findings were consistent with metastatic iGCTs. IHC analysis of the abdominal lesion showed the following: AE1/AE3 (+), CD117 (+), Ki67 (70%), OCT3/4 (+), and sall-4 (+) (Fig. 5). After four courses of platinum-based chemotherapy, the patient was discharged and upgraded to a fair condition.

## Discussion and conclusions

While iGCTs in the pineal gland are more prevalent in the pediatric population, they are still relatively rare, with a low incidence rate of less than 1% of all intracranial tumors [5]. Nevertheless, because postoperative metastasis appeared successively in two recently hospitalized patients, we conducted a literature search in PubMed to confirm whether a VP shunt can lead to tumor metastasis (Table 1). As the histological features of intracranial GCTs are comparable to those of gonadal seminoma, we quickly focused on a retrospective study of 1011 cases of germinoma conducted by Haimovic et al. [6]. All the patients in his study were children who were diagnosed with primary CNS tumors and whose longest survival time reached 32 years. Only 10 patients developed EIM, 2 of whom were diagnosed with GCTs, 6 with neural tube cell tumors, 1 with ependymoma, and 1 with an atypical rod-shaped tumor. Another study by Belongia and Jogal [7, 8] reported that only 0.98% of patients with primary intracranial tumors developed EIM. In addition, Kun et al. [9] recently reported 2 cases of pineal tumors. Consistent with the previous cases [5], these authors demonstrated the risk of metastasis caused by endoscopic biopsy. Although endoscopic biopsy was not performed in our patients, similar procedures of inserting a tube and collecting excess CSF during placement of a VP shunt were alarming. The possibility of peritoneal implant metastasis by VP shunts in these cases, though uncertain, cannot be denied [10].

**Table 1:** Review of the 18 cases of ventriculoperitoneal shunt-related intra-abdominal metastasis reported between 1979 and 2020 on PubMed

Author	Age	Sex	Histological type	Treatment	Initial position	Metastatic area	VP shunt	Time to metastasis	Initial symptoms	Survival after metastasis
F. Lesoin [10]	4	M	Pineal tumor	Radiotherapy	Posterior part of the 3rd ventricle	Intracranial and abdominal (mesenteric)	Y	12 months	Bilateral, symmetrical, cerebellar syndrome, bilateral, papilledema, Parinaud’s syndrome	10 months
F. Lesoin [10]	3	F	Pineoblastoma	Subtotal resection and radiotherapy	Third ventricle	Behind the bladder, internal mammary glands, subhepatic, and pulmonary	Y	8 months	Character disorders, cerebellar syndrome	>18 months
F. Lesoin [10]	12	M	Pinealoblastoma	Subtotal resection and radiotherapy	Pineal	Pulmonary	Y	12 months	Static, symmetrical, bilateral, cerebellar syndrome	68 months
Belongia and Jogal [7]	7	M	Mixed malignant	Chemotherapy	N/A	Multiple intra-abdominal masses	Y	5 months	Asymptomatic, incidental finding on spinal imaging	10 months
Murray et al. [11]	13	F	Pineal germinoma	Resection of mass and chemotherapy	Pineal	Ascites, pelvic, mass, peritoneal nodules	Y	17 months	Abdominal distension	>34 months
Altundag et al. [1]	23	M	Pineal germinoma	Chemotherapy	Pineal	Ascites, pelvic, mass, multiple liver nodules	Y	24 months	Abdominal distension	>36 months
Back et al. [2]	10	M	Pineal germinoma	Chemoradiotherapy and resection of the mass	Pineal	Abdominal mass	Y	13 months	Abdominal pain and distension	>4 months
Ung et al. [12]	13	M	Pineal germinoma	Resection of mass and chemotherapy	Pineal	Abdominal mass	Y	37 months	Abdominal pain	>24 months
Pallini et al. [13]	15	M	Pineal germinoma	Subtotal resection and radiotherapy	Pineal and 3rd ventricle	Pelvic mass, peritoneal nodules	Y	2 months	Headache, lethargy, limitation, upward gaze, diabetes insipidus	4 months
Kim et al. [8]	36	M	Pineal germinoma	Chemotherapy	Pineal	Ascites, peritoneal nodules	Y	12 months	Abdominal distension, vomiting	>6 months
Devkota et al. [5]	12	M	Pineal germinoma	N/A	Pineal	Pelvic mass, peritoneal nodules	Y	24 months	Abdominal pain, distension, vomiting	<1 month
Haimovic et al. [6]	27	M	Pineal germinoma	Radiotherapy	Posterior 3rd ventricle	right abdominal palpable rectally	Y	36 months	Bifrontal, headaches, diplopia, nausea, dizziness, hydrocephalus, duration	NA
Kun et al. [9]	14	M	Germinoma	Radiotherapy	NA	Pelvic mass	Y	14 months	Abdominal pain, constipation	>38 months
Triolo and Schulz [14]	15	M	Pineal germinoma	N/A	Pineal	Pelvic abdominal mass, peritoneal and omental nodules	Y	N/A	Constipation, weight loss	21 months
Wood et al. [15]	11	M	Pineal germinoma	Chemoradiotherapy	Pineal	Pelvic mass	Y	36 months	Rectal discomfort	>24 months
Wood et al. [15]	13	F	Pineal germinoma	N/A	Pineal	Pelvic mass	Y	10 months	N/A	<1 month
Wood et al. [15]	15	M	Pineal germinoma	Chemotherapy	Pineal	Fluid collection	Y	36 months	Abdominal pain distension	N/A

Although abdominal metastasis is a low-probability event, the two patients described above were treated with total central radiotherapy, which poses a challenge for subsequent treatment after abdominal metastasis. We believe that chemotherapy alone is far from sufficient for germ cell tumors. Radiotherapy is an important way to improve prognosis under the premise of inoperability. However, since patients undergo whole-CNS radiotherapy, the spinal cord and bilateral kidneys are irradiated with different doses; therefore, whether there is an opportunity for whole-abdominal radiotherapy needs to be assessed according to the first radiotherapy plan. Given the pathogenetic process, history, and clinical and imaging manifestations, which are entirely in line with the primary diagnosis and standard treatment, their diagnoses were highly likely to be GCTs. Patients receiving the standard regimen (a VP shunt followed by chemoradiotherapy) rarely develop EIM after treatment, but the two patients developed extensive peritoneal metastasis 8 and 32 months after placement of VP shunts. Therefore, we aimed to determine whether tumor cells can be metastasized through a shunt. Unfortunately, cytological analysis of the CSF specimen of the first case was not performed after surgery, but the pathological report of the second case revealed metastatic GCTs. Through previous studies reported in the literature, we found that although the placement of a VP shunt is a classic surgical procedure for the emergency relief of hydrocephalus, it still has a risk of metastasis. Therefore, for tumors with a high risk of dissipating, the third ventricle fistula may be a better choice, which not only relieves hydrocephalus but also avoids the maintenance of the shunt tube and the risk of abdominal dissemination in the later stage. Moreover, if conditions permit, neurosurgeons have an opportunity to perform a biopsy to obtain a clear pathology.

Intracranial GCTs are rare and rarely progress to distant metastases; however, peritoneal implant metastasis occurred in two cases 8 and 32 months after placement of VP shunts. As Devkota et al. [5] stated that GCTs could metastasize along the endoscopic biopsy pathway, we believe that peritoneal metastases are likely to be disseminated from the shunt pathway.

Regarding the metastatic pathway of the two cases reported herein, we believe that GCTs are likely to metastasize through the CSF and have a high possibility of developing distant metastasis along the VP shunt pathway.

## Supplementary Information

**Additional file 1: Fig. S1.** MRI of the central nervous system showed no evidence of tumor recurrence in the second patient.

**Additional file 2: Fig. S2.** Intraoperative resection of the lesion confirmed the diagnosis of mixed germ cell tumor.

## Data Availability

Not applicable.

## Abbreviations

CNS GCTs: Central nervous system germ cell tumors
WHO: World Health Organization
NGGCTs: Non-germinal GCTs
HIP: High intracranial pressure
CSF: Cerebrospinal fluid
AFP: Alpha-fetoprotein
β-HCG: Chorionic gonadotropin β
VP: Ventriculoperitoneal
IHC: Immunohistochemical
18F-FDG PET/CT: 18-Fluorodeoxyglucose positron emission tomography/computed tomography
EIM: Extensive intraperitoneal metastasis
