# Supplementary figures and images for: The external metastasis of the central nerve system germ cell tumors: case report and review of the literature
Source: Chin Neurosurg J. 2021 Jun 2;7:29. doi: 10.1186/s41016-021-00246-0 (PMC8170731; doi:10.1186/s41016-021-00246-0)

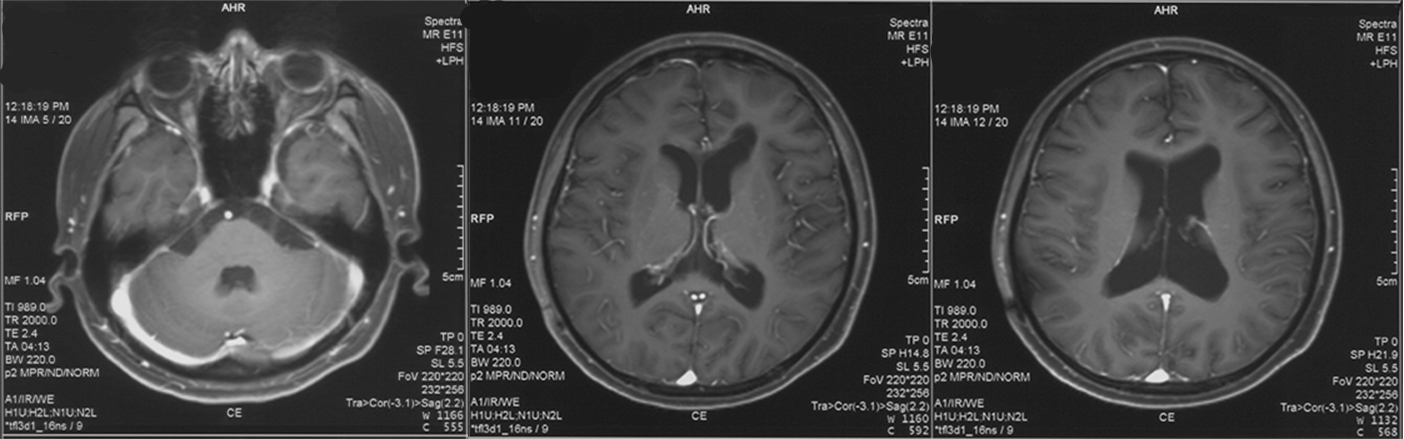

Supplement: Supplementary file 1 — Additional file 1: Fig. S1. MRI of the central nervous system showed no evidence of tumor recurrence in the second patient. [file 41016_2021_246_MOESM1_ESM.tiff]

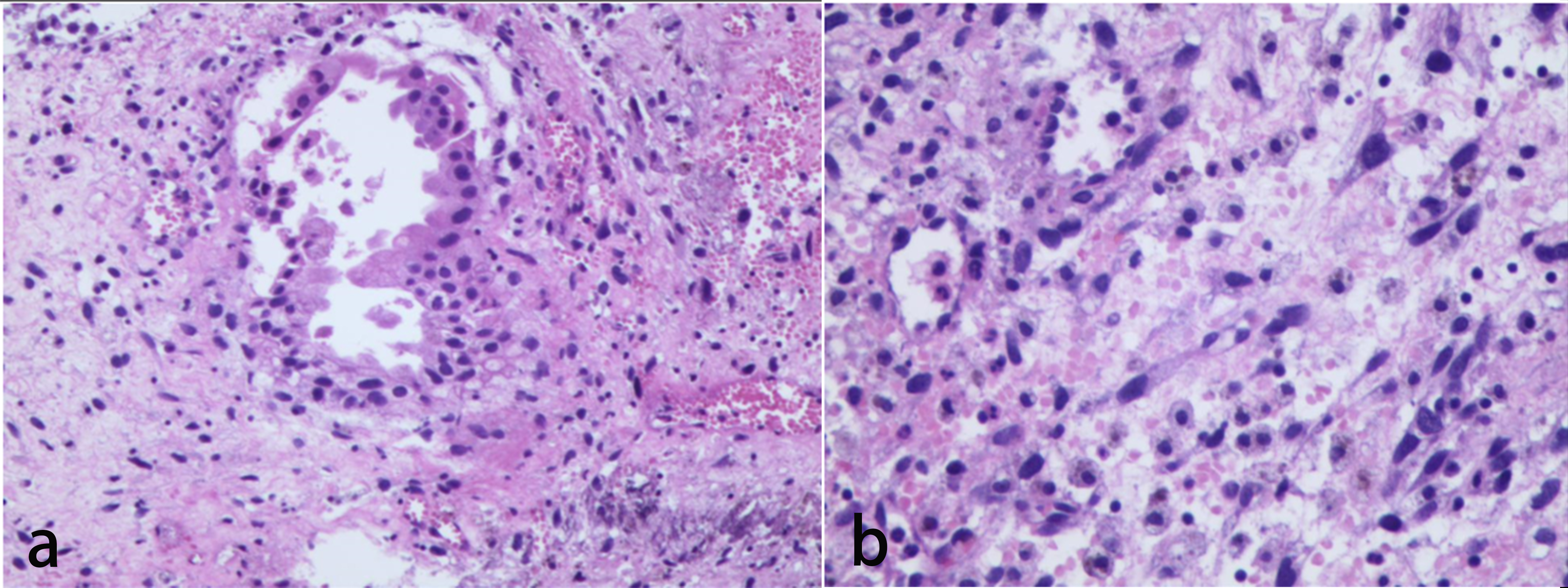

Supplement: Supplementary file 2 — Additional file 2: Fig. S2. Intraoperative resection of the lesion confirmed the diagnosis of mixed germ cell tumor. [file 41016_2021_246_MOESM2_ESM.tiff]
